# Association of Two Variants in *SMAD7* with the Risk of Congenital Heart Disease in the Han Chinese Population

**DOI:** 10.1371/journal.pone.0072423

**Published:** 2013-09-05

**Authors:** Erli Wang, Wenfei Jin, Wenyuan Duan, Bin Qiao, Shuna Sun, Guoying Huang, Kaihu Shi, Li Jin, Hongyan Wang

**Affiliations:** 1 Chinese Academy of Sciences and Max Planck Society (CAS-MPG) Partner Institute for Computational Biology, Shanghai Institutes for Biological Sciences, Chinese Academy of Sciences, Shanghai, China; 2 The State Key Laboratory of Genetic Engineering and Ministry of Education (MOE) Key Laboratory of Contemporary Anthropology, School of Life Sciences, Fudan University, Shanghai, China; 3 Institute of Cardiovascular Disease, General Hospital of Jinan Military Region, Jinan, China; 4 Children's Hospital of Fudan University, Shanghai, China; 5 Second Hospital of Anhui Medical University, Hefei, China; 6 The Institutes of Biomedical Sciences, Fudan University, Shanghai, China; Leiden University Medical Center, The Netherlands

## Abstract

SMAD7 is a general antagonist of TGF-β signaling and has been found to be involved in cardiogenesis in mouse models, but its role in human congenital heart disease (CHD) has yet to be investigated. To examine if *SMAD7* is associated with CHD, we conducted a case-control study in the Han Chinese population. Exon1 and exon4 of *SMAD7*, which encode the functional MH1 and MH2 domains, were directly sequenced in 1,201 sporadic CHD patients and 1,116 control individuals. A total of 18 sequence variations were identified. Two common variants rs3809922 and rs3809923 are located at exon4 of SMAD7, and were found in strong linkage disequilibrium with each other (*r* ^2^ = 0.93). We analyzed the association of these two loci with CHD in 3 independent subgroup case-control studies, and found that in some subgroups, rs3809922 and rs3809923 were significantly associated with CHD through genetic model analysis. In the combined data set, TT genotype in rs3809922 significantly increased the risk of CHD compared with CC and CT, while GG genotype in rs3809923 significantly increased the risk of CHD compared with CC and CG, particularly in the recessive model. In addition, haplotype analyses showed that haplotype TG significantly increased the risk of CHD (*P* = 6.9×10^−6^); this finding supports the results from the analyses based on single locus. According to data from the 1000 Genomes Project, the frequencies of the two risk alleles varied greatly between populations worldwide, which indicate the identified associations might have a population difference. To our knowledge, this is the first report that genetic variants in *SMAD7* influence susceptibility to CHD risk.

## Introduction

Cardiac morphogenesis involves acute spatial and temporal regulation of numerous factors and signal pathways. Any disturbance to this finely regulated, complex process may lead to the occurrence of heart defects [1]. Congenital heart disease (CHD) is one of the most common birth defects worldwide and is the leading cause of mortality and morbidity in newborns. CHD occurs in approximately 1–8% of live births and is responsible for approximately 10% of infant deaths [1]–[3]. Great efforts have been made during the past decade to elucidate the pathogenesis of CHD. It is generally accepted that CHD has a genetic component and that environmental factors also contribute to disease etiology [4], [5]; however, the underlying mechanisms remain largely obscure.

SMAD7 is a nuclear transcription factor induced by TGF-β and acts as an intracellular inhibitor of TGF-β signaling through many mechanisms via a negative feedback loop [6], [7]. In addition, SMAD7 acts as a mediator of cross-talk between TGF-β and other signaling pathways [8]. These processes are finely regulated, and abnormal expression of SMAD7 has been proved to affect a variety of human diseases, such as various tumorigenesis, tissue fibrosis, and intestinal inflammation [9]–[14].

SMAD7 contains two functional mad homology (MH) domains linked by a non-conserved linker region. The MH1 domain is located at the N-terminus and the MH2 domain is located at the C-terminus. Truncated SMAD7 lacking the C-terminal MH2 domain is unable to promote myogenic differentiation [15]. Studies of mice deficient in Smad7 demonstrated the critical roles this protein played in heart homeostasis. Chen *et al*. reported that most Smad7 homozygous mutant mice lacking the MH2 domain died *in utero* due to severe defects in cardiovascular development, such as ventricular septal defects and outflow tract malformation; however, deletion of exon1 did not manifest a different heart phenotype [16]. Over-expression of Smad7 also results in congenital cardiovascular defects [17]. Therefore, SMAD7 is essential for cardiac development. It is assumed that SMAD7, especially the MH2 domain, is associated with an increased risk of human CHD, but no research has been conducted to explore this hypothesis as yet.

Ongoing researches demonstrate that variations in genes contribute to the occurrence of CHD to a large degree [4]. Thus, we carried out a case-control study in a large Han Chinese population (*n* = 2,317) to examine if *SMAD7* was associated with CHD. We found that two highly linked SNPs, rs3809922 and rs3809923, were associated with an increased risk of CHD, especially with septation defects, the main CHD subtype. These results established common genetic variations of *SMAD7* as susceptibility loci for CHD for the first time and further analysis indicated that the association might be population specific. Our study provided new insight into the development of congenital heart defects.

## Materials and Methods

### Ethics Statement

Written informed consent from the parents or guardians of the children was obtained. Protocols used in this work were reviewed and approved by the Ethics Committee of the School of Life Sciences, Fudan University prior to the commencement of the study.

### Study population

Samples from a total of 1,201 CHD patients (mean age 6.5±8.3 years, 48.3% female) were collected from different regions across China, between August 2008 and May 2011. Of these, 601 CHD samples were from the Cardiovascular Disease Institute of Jinan Military Command (Jinan, Shandong Province, China), 456 were from the Children's Hospital of Fudan University (Shanghai, China), and 144 were from the Second Hospital of Anhui Medical University (Hefei, Anhui province, China). Routine clinical diagnoses were conducted, and patients with a variety of CHD symptoms were recruited while individuals with syndromes were excluded. The age at diagnosis ranged from a few hours after birth to the teens. Detailed diagnosis information for the patients was shown in Table S1. Septation defects are the most frequently occurring type of CHD. Notably, ventricular septal defect (VSD) is the most common subtype of CHD in China and accounts for approximately 42% of the total cases in our study. The 1,116 controls, (mean age 5.9±9.7 years, 42.2% female) with no heart related or other serious diseases, were recruited from the same geographic area during the same time period and were matched with the CHD cases for age and sex. All subjects were genetically ethnic Han Chinese.

### Direct sequencing of human *SMAD7* gene in the coding regions

**Figure 1:**
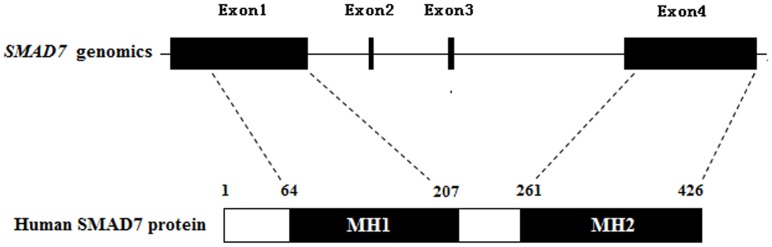
Schematic of *SMAD7* and the encoded protein. MH1, MAD homology 1 domain; MH2, MAD homology 2 domain.

The human *SMAD7* gene (NM_005904.3), mapping to 18q21.1, contains 4 exons and encodes a nuclear protein with 426 amino acids (Figure 1). Exon2 and exon3 of *SMAD7* encode the non-conserved linker region, while exon1 and exon4 encode the conserved MH1 and MH2 functional domains which are pivotal for specific inhibition of TGF-β signaling. Therefore, we selected exon1 and exon4 as the target for sequencing.

Whole blood was collected from each study individual. Genomic DNA was extracted using standard methods and quantified using a NanoDrop2000 (Thermo Scientific, Wilmington, DE, USA). Exon1 and exon4 of *SMAD7* were amplified by polymerase chain reaction (PCR). The online software Primer3 (v. 0.4.0) was used to design the PCR primers. Primers are listed in Table S2 and reaction conditions are available upon request. BigDye Terminator v3.1 was used for the direct sequencing reaction according to the manufacturer's instructions (Applied Biosystems, Carlsbad, CA, USA). Samples were processed on an automated 3730 sequencer (Applied Biosystems, Carlsbad, CA, USA), and the results were analyzed using Sequence Scanner v1.0 and DNAStar software.

### Statistical analysis

We used R (version 2.13) and PLINK [18] to conduct most of the statistical analyses in this study. χ^2^ test was performed to assess Hardy-Weinberg Equilibrium (HWE). We applied the commonly used statistic tests in this study in order to find the best model to explain the genetic mechanism. We evaluated the association of each variant in *SMAD7* with CHD via five genetic models of inheritance, including recessive, dominant, multiplicative (allelic), genotypic and additive models [19]. In the recessive model, individuals that were homozygous for risk alleles were coded as 1, while other genotypes were coded as 0. In the dominant model, individuals carrying at least one risk allele were coded as 1, and those who were homozygous for two non-risk alleles were coded as 0. In the multiplicative model, the total number of risk and non-risk alleles was compared between cases and controls, regardless of the genotypes of the individuals carrying the alleles. The genotypic model treated each genotype with different susceptibility, and we coded them as 0, 1 and 2 to represent the number of risk alleles they carried. We used the χ^2^ test to evaluate whether there was significant difference between cases and controls under the recessive, dominant, multiplicative and genotypic models. In the additive model, the increased disease risk of genotype carrying one risk allele is half of that for homozygous of risk alleles, and the Cochran-Armitage test for trend was conducted for evaluating the difference between case and control in this model. We calculated the odds ratios (OR) and 95% confidence intervals (CIs) under each model. PHASE [20] was used to infer the haplotypes, and we conducted an association analysis based on the inferred haplotypes. In the haplotype analyses, we treated the most common haplotype in control as the reference allele. Statistical analyses on the haplotypes were performed in a similar way to that on single locus.

## Results

### Identification of human *SMAD7* variations

We identified 18 genetic variants by direct sequencing of *SMAD7* in a total of 2,317 individuals, including 1,201 patients with pathologically confirmed CHD and 1,116 matched control subjects (Table 1). Two synonymous polymorphisms, rs3809922 (c.894C>T, p.L298L) and rs3809923 (c.1206C>G, p.G402G), were common variants with minor allele frequencies (MAF) greater than 0.1 in both cases and controls. The other variations in the coding region were rare mutations (MAF<0.005), including five synonymous mutations and one non-synonymous mutation only found in the CHD population, three synonymous and five non-synonymous mutations only found in the control population and one synonymous mutation found in both case and control populations. A variant in the 3′UTR (rs181999754) adjacent to exon4 was also identified in both CHD and control populations. Multiple alignment of the six non-synonymous mutations identified in *SMAD7* showed that these mutations were conserved in mammals (Figure S1).

**Table 1:** *SMAD7* sequence variants identified in CHD and control subjects.

Location	Nucleotide change	Amino acid change	Protein domain	Patients^a^ (n = 1201)	Controls^a^ (n = 1116)	Novel /reported
Non-synonymous						
Exon1	c.224C>T	p.P75L	MH1	0	1	Novel
	c.403A>G	p.R135G	MH1	0	2	Novel
	c.560A>G	p.N187S	MH1	0	1	Novel
Exon4	c.1084C>T	p.P362S	MH2	0	1	Novel
	c.1172C>T	p.T391M	MH2	0	1	Novel
	c.1247C>A	p.P416Q	MH2	1	0	Novel
Synonymous						
Exon1	c.108G>A	p.E36E		0	1	Novel
	c.147G>A	p.G49G		1	0	rs181999754
	c.183C>T	p.C61C		0	3	Novel
	c.312G>A	p.K104K	MH1	2	2	Novel
Exon4	c.894C>T	p.L298L	MH2	308/41^b^	281/19^b^	rs3809922
	c.951C>T	p.I317I	MH2	1	0	Novel
	c.1062G>A	p.T354T	MH2	1	0	Novel
	c.1122G>A	p.A374A	MH2	1	0	Novel
	c.1173G>A	p.T391T	MH2	0	1	rs34151545
	c.1206C>G	p.G402G	MH2	317/47^b^	292/20^b^	rs3809923
	c.1218C>T	p.C406C	MH2	1	0	Novel
3′UTR	c.1281+23G>A			8	7	rs181999754

a These columns indicate the number of heterozygotes. Where there are two numbers, the first represents the number of heterozygotes, and the second represents the number of homozygotes.

b Common variations with minor allele frequencies (MAF) ≥10%; others variations are rare mutations with MAF <1%.

### Statistical result

Here, we focused on analysis of the two common variants, rs3809922 and rs3809923. They were located in exon4 of *SMAD7*, which encodes the MH2 functional domain. These two loci were in strong linkage disequilibrium (LD) with each other according to the sequencing result (*r* ^2^ = 0.93) and were both in Hardy-Weinberg Equilibrium in the control population (Table S3).

We firstly analyzed the risk association of rs3809922 and rs3809923 with CHD using five different genetic models in 3 independent case-control pairs. The results indicated that, compared with CC and CT, the rs3809922 TT genotype was significantly associated with CHD in both the recessive and genotypic models in Shanghai group, while there was no such association in Shandong and Anhui group (Table 2). Nevertheless, the combined data of the 3 groups lead to increased statistical power and found more significant association, and in particular, the P-values were extremely low in the recessive model in which two copies of T significantly increase the risk of CHD (*P* = 0.0096), with OR 2.04 (95% CI = 1.18–3.52). As for rs3809923, the GG genotype was significantly associated with CHD in the recessive model in both Shandong and Shanghai group. In the combined analysis, the rs3809923 GG genotype, compared with CC and CG, was significantly associated with CHD in the recessive model, as well as the general genotypic, multiplicative and additive models (Table 3). In particular, in the combined data set, two copies of G significantly increase the risk of CHD (*P* = 0.0023), with OR 2.23 (95% CI = 1.32–3.77).

**Table 2:** Association of *SMAD7* rs3809922 variant with CHD in 3 independent case-control studies.

Rs3809922	Genetic model	Pattern	Case	Control	P-value	OR (95% CI)
	Recessive	TT/TC+CC	21/582	11/538	0.127	1.76 (0.85–3.65)
	Genotypic	TT/CT/CC	21/157/425	11/128/410	0.148#	NA
Shandong	Multiplicative	T/C	199/1007	150/948	0.058	1.25 (0.99–1.57)
	Additive	TT/CT/CC	21/157/425	11/128/410	0.062*	NA
	Dominant	TT+TC/CC	178/425	139/410	0.111	1.24 (0.95–1.60)
	Recessive	TT/TC+CC	17/437	7/464	0.031	2.58 (1.09–6.13)
	Genotypic	TT/CT/CC	17/100/337	7/126/338	0.033#	NA
Shanghai	Multiplicative	T/C	134/774	140/802	0.95	0.99 (0.77–1.28)
	Additive	TT/CT/CC	17/100/337	7/126/338	0.95*	NA
	Dominant	TT+TC/CC	117/337	133/338	0.40	0.88(0.66–1.18)
	Recessive	TT/TC+CC	3/141	1/95	0.65§	0.49 (0.01–6.28)
	Genotypic	TT/CT/CC	3/51/90	1/27/68	0.47§	NA
Anhui	Multiplicative	T/C	57/231	29/163	0.19	1.39 (0.85–2.26)
	Additive	TT/CT/CC	3/51/90	1/27/68	0.17*	NA
	Dominant	TT+TC/CC	54/90	28/68	0.23	1.45 (0.84–2.53)
	Recessive	TT/TC+CC	41/1160	19/1097	0.0096	2.04 (1.18–3.52)
	Genotypic	TT/CT/CC	41/308/852	19/281/816	0.031#	NA
Combined	Multiplicative	T/C	390/2012	319/1913	0.066	1.16 (0.99–1.36)
	Additive	TT/CT/CC	41/308/852	19/281/816	0.069*	NA
	Dominant	TT+TC/CC	349/852	300/816	0.24	1.11 (0.93–1.34)

All the P-values were calculated based on the χ^2^ except the ones specifically indicated. OR, odds ratio; CI, confidence interval; NA =  not available;

# , degrees of freedom  = 2;

* , P-value was calculated based on Cochran–Armitage test.

§ , indicated Fisher's exact test.

**Table 3:** Association of *SMAD7* rs3809923 variant with CHD in 3 independent case-control studies.

Rs3809923	Genetic model	Pattern	Case	Control	P-value	OR (95% CI)
	Recessive	GG/GC+CC	25/578	11/538	0.037	2.12 (1.04–4.29)
	Genotypic	GG/CG/CC	25/163/415	11/135/40	0.057#	NA
Shandong	Multiplicative	G/C	213/993	157/941	0.028	1.28 (1.03–1.61)
	Additive	GG/CG/CC	25/163/415	11/135/403	0.031*	NA
	Dominant	GG+GC/CC	188/415	146/403	0.087	1.25 (0.96–1.61)
	Recessive	GG/GC+CC	19/435	7/464	0.013	2.90 (1.23–6.79)
	Genotypic	GG/CG/CC	19/102/333	7/131/333	0.012#	NA
Shanghai	Multiplicative	G/C	140/768	145/797	0.98	1.00 (0.78–1.29)
	Additive	GG/CG/CC	19/102/333	7/131/333	0.98*	NA
	Dominant	GG+GC/CC	121/333	138/333	0.37	0.88 (0.66–1.17)
	Recessive	GG/GC+CC	3/141	2/94	1.00§	1.00 (0.08–8.90)
	Genotypic	GG/CG/CC	3/52/89	2/26/68	0.34§	NA
Anhui	Multiplicative	G/C	58/230	30/162	0.21	1.36 (0.84–2.20)
	Additive	GG/CG/CC	3/52/89	2/26/68	0.19*	NA
	Dominant	GG+GC/CC	55/89	28/68	0.17	0.67 (0.37–1.20)
	Recessive	GG/GC+CC	47/1154	20/1096	0.0023	2.23 (1.32–3.77)
	Genotypic	GG/CG/CC	47/317/837	20/292/804	0.0088#	NA
Combined	Multiplicative	G/C	411/1991	332/1900	0.038	1.18 (1.01–1.38)
	Additive	GG/CG/CC	47/317/837	20/292/804	0.040*	NA
	Dominant	GG+GC/CC	364/837	312/804	0.21	1.12 (0.94–1.34)

All the P-values were calculated based on the χ^2^ except the ones specifically indicated. OR, odds ratio; CI, confidence interval; NA =  not available;

# , degrees of freedom  = 2;

* , P-value was calculated based on Cochran–Armitage test.

§ , indicated Fisher's exact test.

Septation defects, such as ventricular septal defects and atrial septal defects, were the most prevalent subtypes of CHD worldwide [3], and in our samples they account for nearly 70% of the total cases. When the subtypes of CHD were considered, both rs3809922 and rs3809923 were significantly associated with septation defects in the recessive, genotypic, multiplicative and additive models (Table 4). We did not find any risk association between the two loci and other CHD subtypes; this may be partly due to the limited sample size of other CHD subtypes (data are available on request).

**Table 4:** Association of rs3809922 and rs3809923 variants with septation defects.

Rs3809922	Genetic model	Pattern	Case	Control	P-value	OR (95% CI)
	Recessive	TT/TC+CC	27/738	19/1097	0.012	2.11 (1.17–3.80)
	Genotypic	TT/CT/CC	27/210/528	19/281/816	0.017#	NA
rs3809922	Multiplicative	T/C	264/1266	319/1913	0.014	1.25 (1.05–1.49)
	Additive	TT/CT/CC	27/210/528	19/281/816	0.015*	NA
	Dominant	TT+TC/CC	237/528	300/816	0.053	1.22 (1.00–1.49)
	Recessive	GG/GC+CC	33/732	20/1096	0.001	2.47 (1.41–4.31)
	Genotypic	GG/CG/CC	33/223/509	20/292/804	0.001#	NA
rs3809923	Multiplicative	G/C	289/1241	332/1900	0.001	1.33 (1.12–1.58)
	Additive	GG/CG/CC	33/223/509	20/292/804	0.001*	NA
	Dominant	GG+GC/CC	256/509	312/804	0.011	1.29 (1.06–1.58)

All the P-values were calculated based on the χ^2^ test unless otherwise indicated.

# , degrees of freedom  = 2;

* , P-value was calculated based on the Cochran–Armitage test.

In addition, we inferred the haplotypes of the two linked variants (rs3809922 and rs3809923) using PHASE [20]. The result showed that haplotype TG significantly increased the risk of CHD (*P* = 6.9×10^−6^), with OR 2.13 (95% CI = 1.53–2.97) (Table 5). Other haplotypes did not show any association.

**Table 5:** Haplotype analysis of rs3809922 and rs3809923.

Haplotype	Case (Frequency)	Control (Frequency)	OR (95% CI)	P- value
CC**	2250 (0.937)	2155 (0.897)	1.00	NA
CG§	4 (0.002)	4 (0.002)	0.96 (0.18–5.15)	1
TC	32 (0.013)	21 (0.009)	1.46 (0.84–2.52)	0.23
TG	116 (0.048)	52 (0.022)	2.13 (1.53–2.97)	6.9×10^−6^

** , this haplotype was chosen as a reference haplotype due to its high frequency.

§ , Fisher's exact test.

## Discussion

TGF-β signaling mediated by SMADs is critical for a great many cellular and developmental processes and plays a key role in embryogenesis and tissue homeostasis [21]. Recent studies have shown that TGF-β signaling is essential for the function of the cardiovascular system and in particular, SMAD6 has been reported to be associated with human congenital cardiovascular malformation [22]. Smad7 is critical for heart development in mouse models [16], [17], [23], [24]. Therefore, it was hypothesized that SMAD7 might be associated with the risk of congenital heart defects in humans. Here, we provide evidence supporting this assumption.

We performed a large case-control study to test the hypothesis that variants in *SMAD7* are associated with CHD, and initially identified that alleles in two genetic variants of *SMAD7* (rs3809922 and rs3809923) were significantly associated with susceptibility for CHD in the Han Chinese population. Both allele T in rs3809922 and allele G in rs3809923 were risk factors, especially when analyzed with a recessive model (Table 2 and Table 3). The association was more significant when the septation defects subtype was considered (Table 4). Further analysis of the haplotypes showed that the haplotype TG was significantly enriched in CHD cases. This result was essentially consistent with the analysis based on each single locus (Table 5).

Although both of the associated variants were silent polymorphisms that did not alter the encoded SMAD7 protein, the possibility of the variations affecting SMAD7 at the mRNA level could not be ruled out [25]–[27]. Synonymous SNPs may influence the transcriptional efficiency or later stability of mRNA and may ultimately affect protein expression. Previous studies demonstrated that over expression of SMAD7 led to tumorigenesis by disturbing TGF-β induced apoptosis, while deletion of Smad7 increased apoptosis in the hearts of mutant mice [16], [17]. Furthermore, while the majority of homozygous mutant mice with a deletion of the MH2 domain died *in utero* due to various cardiovascular defects (such as VSD and outflow tract malformation), deletion of the MH1 domain of SMAD7 did not cause changes to the cardiovascular phenotype [16]. The MH2 domain of SMAD7 is important for the inhibition of TGF-β signaling [28], and both rs3809922 and rs3809923 are located at the MH2 domain. They may disrupt TGF-β signaling by affecting SMAD7 expression, thus impairing normal cardiac development and contributing to CHD predisposition.

**Figure 2:**
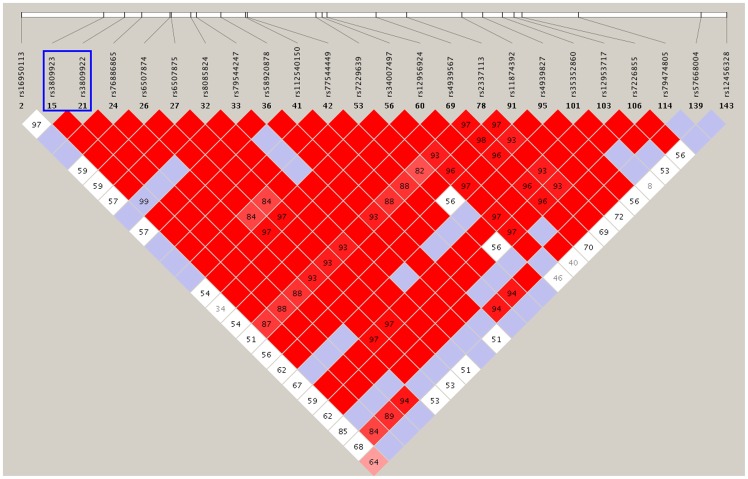
A region that was in extremely strong LD with rs3809922 and rs3809923. Each box represents the LD relationship between two SNPs. The strength of the LD was shown in increasing of red for higher LOD score which indicated higher LD. The white line on the top represents the relative physical position of the SNPs on the chromosome. The value in each box was the |D'| (×100) between each pair of SNPs, another statistics to measure LD.

Alternatively, these variants may not cause the disease themselves but may be in LD with other or unknown disease-causing variations in the regulatory region of *SMAD7*. For example, a novel variant mapping to the enhancer of *SMAD7* was found to be in LD with the colorectal cancer risk variant rs4939827 (intron 3) identified in a GWAS study; this variant proved to be functional in transcription factor binding and gene expression [9], [11]. Furthermore, because single variant is unlikely to have a large impact on the expression of a transcript, gene variants may increase disease risk by interacting with other potential risk alleles. We used haploview [29] to infer regions in strong LD with rs3809922 and rs3809923 based on Han Chinese data from the 1000 Genomes Project. The genomic regions were extended approximately 50 Kb around the two variants (Figure S2). We found an approximately 10 Kb region that was in extremely strong LD with the two risk alleles (Figure 2). Future challenges will be to determine whether the associated SNPs play functional roles in susceptibility to CHD and elucidate the mechanism by which genetic variants influence CHD risk.

We also analyzed the variants status of rs3809922 and rs3809923 based on data from the 1000 Genomes Project. The result showed that the allele frequencies of these two variants varied greatly in different populations (Table 6). In European and Africa populations, the risk allele frequencies were particularly low or the loci were monomorphic. However, the frequencies of the two risk alleles were comparatively high in East Asian population, and the risk allele frequencies of CHB (Han Chinese in Beijing, China) and CHS (Han Chinese South) populations are similar to the result observed in the control population in this study (Table 6 and Table S3). Given this finding, we hypothesized that there might be population or regional differences in susceptibility for CHD for rs3809922 and rs3809923 variants.

**Table 6:** Ancestral allele frequency of rs3809922 C and rs3809923 C in different populations based on data from the 1000 Genomes Project.

	Population*	Rs3809922	Rs3809923
European	CEU	1.0000	1.0000
	FIN	0.9946	0.9946
	GBR	1.0000	1.0000
	IBS	1.0000	1.0000
East Asian	CHB	0.8763	0.8660
	CHS	0.8650	0.8700
	JPT	0.9045	0.9101
West African	LWK	1.0000	1.0000
	YRI	1.0000	1.0000
Americas	ASW	0.9661	0.9746
	CLM	0.9083	0.9083
	MXL	0.9242	0.9242
	PUR	0.9727	0.9636

* The detailed information of these populations can be found at the website: http://www.1000genomes.org/about.

In summary, by direct sequencing of *SMAD7* in a large Han Chinese population, we identified two coding sequence variants which contributed to CHD risk. Our study linked variations in the TGF-β signaling component to human cardiovascular defects, and provides evidence for a ‘common-disease common-variant’ model of CHD predisposition. Identification of these risk loci provides a new perspective on CHD causation. Considering the important role of SMAD7 in cardiac development in early embryos and normal cardiac function in adults, investigation of the mechanisms by which these genetic variants affect CHD risk could provide opportunities to develop new diagnostic and therapeutic strategies.

## Supporting Information

Figure S1 **Sequence alignment of SMAD7 non-synonymous mutations identified in this study.**
(TIF)

Figure S2 **Linkage analysis around the two risk alleles (rs3809922 and rs3809923) which were** **extended about 50 Kb.**
(TIF)

Table S1 **Phenotypes of screened population with CHD.**
(DOC)

Table S2 ***SMAD7*** **primers used in PCR amplification.**
(DOC)

Table S3 **Genotype frequencies of** ***SMAD7*** **rs3809922 and rs3809923 in 1,201 CHD patients and 1,116 controls.**
(DOC)
