# Supplementary figures and images for: Association of Two Variants in SMAD7 with the Risk of Congenital Heart Disease in the Han Chinese Population
Source: PLoS One. 2013 Sep 5;8(9):e72423. doi: 10.1371/journal.pone.0072423 (PMC3764115; doi:10.1371/journal.pone.0072423)

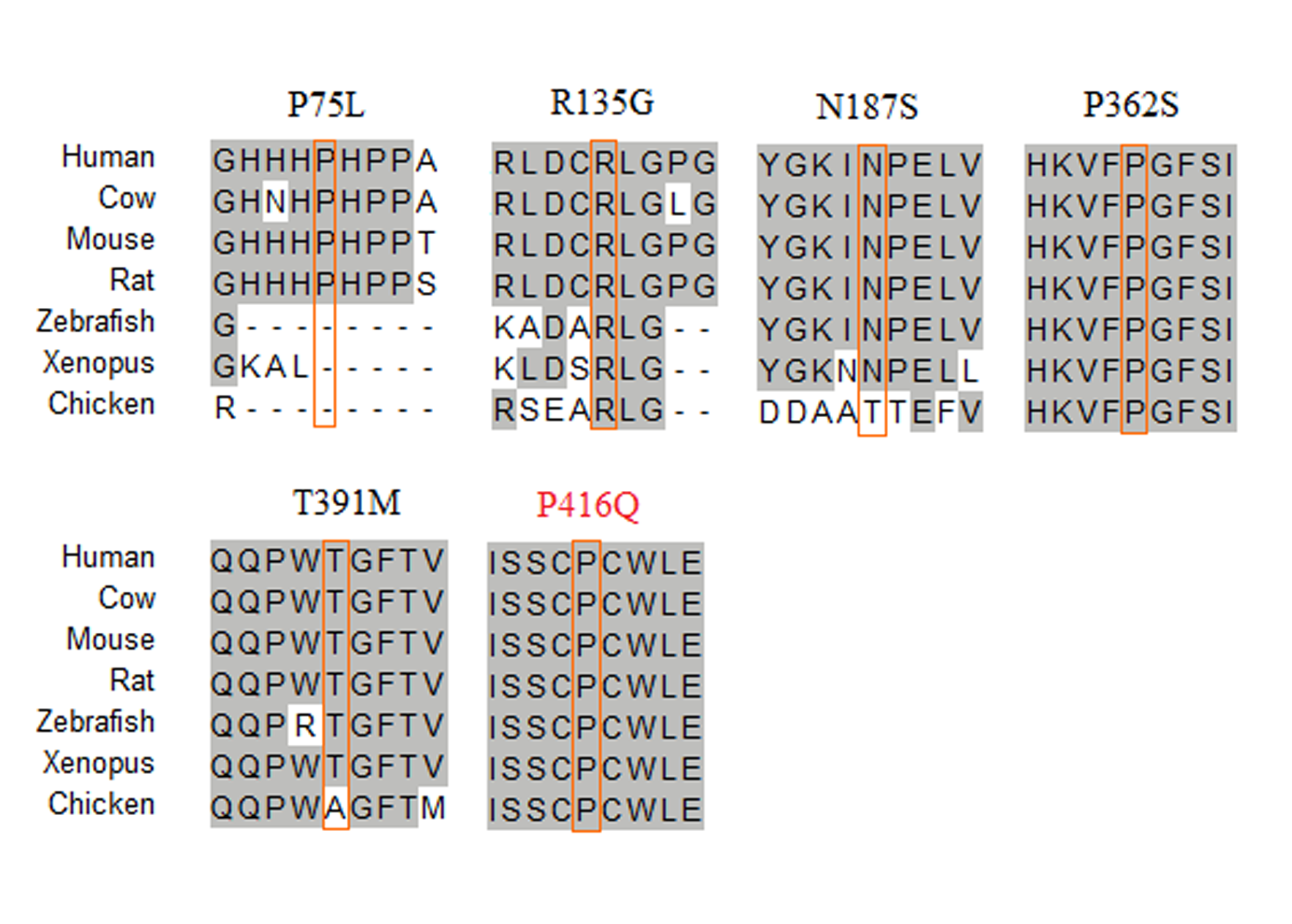

Supplement: Figure S1 — Sequence alignment of SMAD7 non-synonymous mutations identified in this study. (TIF) [file pone.0072423.s001.tif]

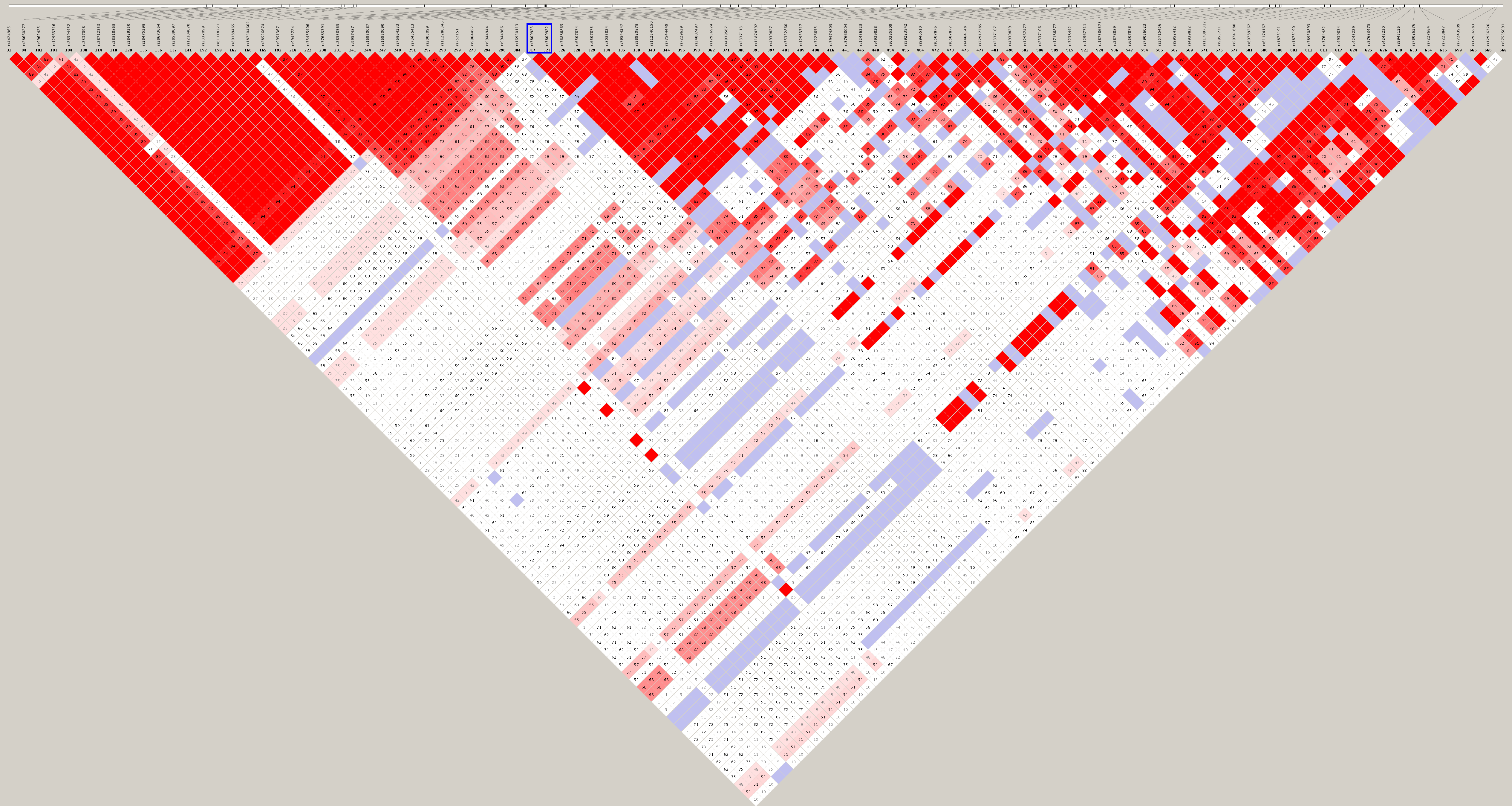

Supplement: Figure S2 — Linkage analysis around the two risk alleles (rs3809922 and rs3809923) which were extended about 50 Kb. (TIF) [file pone.0072423.s002.tif]
