# Supplementary material for: Association of Two Variants in SMAD7 with the Risk of Congenital Heart Disease in the Han Chinese Population
Source: PLoS One. 2013 Sep 5;8(9):e72423. doi: 10.1371/journal.pone.0072423 (PMC3764115; doi:10.1371/journal.pone.0072423)
Supplement: Table S1 — Phenotypes of screened population with CHD. (DOC) [file pone.0072423.s003.doc]

**Table S1.** Phenotypes of Screened Population with Congenital Heart Defects

| Cardiac diagnosis | Number |
| --- | --- |
| Septation defects  ASD  VSD  ASD/VSD  AVSD  Cyanotic heart disease  TOF  DORV  PVC  PTA  Ebstein’s anomaly  Left-sided obstructive lesions  Mitral stenosis  Aortic coarctation | 765  164  508  82  11  214  150  28  20  14  2  24  14  10 |
| Other types of CHD  PDA  PS  PFO  Others | 119  28  15  36 |

ASD, atrial septal defect; VSD, ventricular septal defect; TOF, tetralogy of Fallot; PDA，paten ductus arteriosus; DORV, double outlet right ventricle;PTA, persistent truncus arteriosus; Others, CHD but subtypes unclear
