# Supplementary material for: Association of Two Variants in SMAD7 with the Risk of Congenital Heart Disease in the Han Chinese Population
Source: PLoS One. 2013 Sep 5;8(9):e72423. doi: 10.1371/journal.pone.0072423 (PMC3764115; doi:10.1371/journal.pone.0072423)
Supplement: Table S2 — SMAD7 primers used in PCR amplification. (DOC) [file pone.0072423.s004.doc]

**Table S2. *SMAD7*** primers used in PCR amplification

| Exon | Forward (5’ to 3’) | Reverse (5’ to 3’) | Product length |
| --- | --- | --- | --- |
| Exon1  Exon4 | CCTAGGGGcttttcttccag  AGTTTGAAGTGTGGCCTGCT | ACTTTTCTCCTCGCCTCCTC  CCCTCTCTGCCAATGTGTTT | 706  614 |
