# Supplementary material for: Association of Two Variants in SMAD7 with the Risk of Congenital Heart Disease in the Han Chinese Population
Source: PLoS One. 2013 Sep 5;8(9):e72423. doi: 10.1371/journal.pone.0072423 (PMC3764115; doi:10.1371/journal.pone.0072423)
Supplement: Table S3 — Genotype frequencies of SMAD7 rs3809922 and rs3809923 in 1,201 CHD patients and 1,116 controls. (DOC) [file pone.0072423.s005.doc]

**Table S3. Genotype frequencies of *SMAD7* rs3809922 and rs3809923 in 1,201 CHD patients and 1,**116 controls

| SNP | Genotype | Patient (n=1201) | Control (n=1116) | MAF | | HWE test *P* ^＆^ | |
| --- | --- | --- | --- | --- | --- | --- | --- |
|  |  |  |  | Cases Controls | |  |  |
| rs3809922 | CC  CT  TT | 852  308  41 | 816  281  19 | 16.2% | 14.3% | | 0.35 |
| rs3809923 | CC  CG  GG | 837  317  47 | 804  292  20 | 17.1% | 14.9% | | 0.27 |

*^＆^P* value for Hardy-Weinberg equilibrium test (HWE) in the control subjects;

MAF, minor allele frequency.
